# What role do self-regulatory mechanisms play in the risky use of digital media by adolescents?

**DOI:** 10.3389/fpsyg.2025.1675778

**Published:** 2025-12-18

**Authors:** Karla Hrbackova, Jakub Hladík, Anna Petr Safrankova

**Affiliations:** Department of Pedagogical Sciences, Faculty of Humanities, Tomas Bata University in Zlín, Zlín, Czechia

**Keywords:** self-regulation, self-control, sense of control, digital media use, screen time, impulsivity, adolescents, risky behavior

## Abstract

As the time spent on digital media usage (DMU) has increased in recent years, there has been growing concern about problematic usage. The overuse of digital media affects up to a quarter of adolescents, although excessive DMU is not always associated with problematic use, a defining feature of which may be a loss of self-control. This study aims to clarify how self-regulatory mechanisms, such as trait self-control, sense of control, and self-regulation of digital media use, are related to risky DMU (excessive internet use and impulsive DMU) as well as time spent on DMU. We draw on a representative sample of 2,697 adolescents aged 11–15 years (mean age = 12.9) attending grade 2 primary schools. Our results show that adolescents with higher levels of self-control as well as a greater sense of control exhibit lower levels of risk-taking (excessive internet use and impulsive digital media use and screen time). Furthermore, increased levels of self-control as well as a greater sense of control predict higher levels of self-regulation in digital media contexts. The findings of this study provide insights regarding the importance of strengthening self-control, especially for at-risk users, and offer a perspective on the role of self-regulatory mechanisms in managing digital behavior. These factors can significantly contribute to better self-regulation of adolescents' digital behavior.

## Introduction

In recent decades, time spent on digital media has increased exponentially (Twenge et al., 2019; Madigan et al., 2022). It is thus essential to consider digital media as an important social context within the lives and development of adolescents alongside such familiar contexts as family, peers and school (Subrahmanyam and Smahel, 2010). Despite the documented positive consequences of digital media (i.e., the development of digital skills, enhanced access to information, social support etc.; Haddock et al., 2022; Shao et al., 2024), excessive use can become problematic under certain conditions. Shapira et al. (2000) describe problematic online behavior as the particular inability of an individual to maintain control over his/her behavior. This inability to control the use of digital media (Billieux and Van der Linden, 2012) can be manifest at the psychological, social, academic and/or professional level (cf. Beard and Wolf, 2001). Excessive digital media usage (DMU) should be viewed in terms of the quantity of time and quality of time spent on digital media (or the degree of negative impacts of DMU; Billieux et al., 2017). Although screen time has traditionally been considered a key indicator of digital media use, empirical studies suggest that the relationship between the two is weak and often non-linear (Przybylski and Weinstein, 2017). This highlights the importance of examining self-regulatory mechanisms that could provide a more nuanced and context-sensitive understanding of adolescents' digital media use (Howard et al., 2025).

Turel and Qahri-Saremi (2016) consider problem digital behavior in terms of the disparity between strong cognitive-emotional bias and weak cognitive-behavioral control. This increased vulnerability is rooted in the asynchronous maturation of two distinct neurobiological systems during adolescence. Specifically, the socio-emotional system matures earlier than the cognitive control system (Marciano et al., 2025; Steinberg, 2010). This dynamic contributes to adolescents becoming significant users of digital media, while also increasing their vulnerability to excessive use and its negative effects.

Thus, through the process of structural maturation, characterized by the gradual integration of the cognitive control system with areas of the socio-emotional system (Steinberg, 2010), a greater degree of self-regulation and impulse control is developed (Suchá et al., 2024).

While a number of studies have examined the importance of self-regulatory mechanisms in the context of digital media use, research in this area among adolescents, who are at a critical stage in the maturation of their self-regulatory systems (Steinberg, 2008), is limited. Currently, there is insufficient research into how self-regulatory mechanisms, including general self-control, subjective sense of control, and domain-specific self-regulation, manifest in specific forms of risky digital behavior, such as excessive and impulsive digital media use. This study extends existing knowledge by focusing exclusively on adolescents and the integration of these three mechanisms. Specifically, we examine the relationships between these mechanisms and risky digital behavior (Screen Time, excessive and impulsive use) and compare the prevalence of these mechanisms in groups of adolescents with varying levels of excessive use. A key contribution is verifying the predictive power of general factors (self-control and sense of control) regarding domain-specific self-regulation in the context of digital media. This approach contributes to a deeper, more integrated understanding of the influence of self-regulatory mechanisms on the digital behavior of adolescents.

## Self-regulation in digital media context

Based on the wide range of DMU, here we use the term “digital behavior.” This term represents all activities that an individual may perform in a digital environment using digital media, e.g., using the internet through a smartphone or other digital device, following and interacting with social media, watching and listening to videos, podcasts, etc. or playing digital games (Wearesocial.com, 2023). Research shows that excessive DMU can be associated with negative psychological and physical consequences (World Health Organization, 2019), and can affect identity formation in adolescents (Iwasa et al., 2023) as well as mental health (Abi-Jaoude et al., 2020). Digital media impacts social relationships within peer groups and family, and can be associated with feelings of loneliness and depression, low self-esteem, low academic achievement or even dropping out of school, along with other behavioral problems (Frost and Rickwood, 2017; Tokunaga and Rains, 2010).

Recent meta-analytic evidence supports these associations, indicating that problematic social media use is consistently related to increased levels of depression, anxiety, sleep disturbances, and reduced overall wellbeing, with these associations moderated by age, gender, and methodological factors such as measurement instruments (Ahmed et al., 2024; Montag et al., 2024).

Research (Restrepo et al., 2020) confirms the elevated level of potential vulnerability of children and adolescents in the context of DMU. During adolescence, individuals become increasingly sensitive to social and emotional rewards, while the centers responsible for impulse control and decision-making have not yet fully developed (King and Delfabbro, 2018). Steinberg (2008) further specifies that this period as the socio-emotional system is developing until the full maturation of the cognitive control system is one in which the individual is highly vulnerable to impulsive risk behaviors (cf. Dodge and Albert, 2012). This developmental imbalance can be better understood through dual-process theory. The theory distinguish between fast, automatic, emotionally driven responses (System 1) and slower, more controlled, cognitive processes (System 2; Kahneman, 2011; Steinberg, 2008). During adolescence, the dominance of System 1 over System 2, which is still maturing, contributes to heightened impulsivity and risk-taking behaviors, including the risky digital media use (Steinberg, 2010). In situations where strong impulsive motives prevail (e.g. the urge to respond immediately to a message) System 1 temporarily dominates System 2 (Zahrai et al., 2022; Soror et al., 2015). As a result, behavior is guided more by automatic impulses than by deliberate self-control (Strack and Deutsch, 2004). Impulsivity is characterized by a tendency toward unplanned action and risk-taking, typically associated with poor planning and quick decision-making (Eysenck and Eysenck, 1977). In a digital context, individuals exhibiting higher levels of impulsivity are often observed among groups characterized by problematic digital behavior. For example, adolescents meeting Young's criteria for ‘internet addiction' showed significantly increased impulsivity compared to control groups (Cao et al., 2007; Young, 1998). Furthermore, research has shown a positive correlation between impulsivity and problematic internet behavior (Mottram and Fleming, 2009), online gambling (Billieux et al., 2015), and smartphone addiction (Salvarli and Griffiths, 2022). Impulsive use can be termed problematic at the point at which the individual can no longer control it (Doebel et al., 2017). Within the dual-process framework, self-control and self-regulation can be understood as cognitive mechanisms associated with the reflective, deliberative processes of System 2. These mechanisms serve to counterbalance the automatic impulses of System 1 (Kahneman, 2011; Steinberg, 2008). Baumeister et al. (2018) define self-control as the ability to initiate action, but also to suppress or resist short-term temptations, urges, impulses, and conflicting tendencies of immediate gratification in order to achieve set goals. Self-control is typically described as the conscious overcoming of a proximal impulse or temptation that is in conflict with the individual's set goals (cf. Leduc-Cummings et al., 2022). Self-control in this case has an intentional inhibitory character (Bauer and Baumeister, 2013). In addition to the individual's ability to resist or suppress impulsive temptations for immediate gratification, active behavioral management toward long-term goals (self-regulation) in the direction of reducing the frequency and intensity of strong impulses should be pursued in the context of digital behavior (Błachnio and Przepiorka, 2016). Self-regulation in this context is seen as the ability to control and change by adjusting emotions and behavior in an adaptive direction (Baumeister et al., 2018). Self-regulation is thus closely related to integral goals, usually long-term ones. In the case of failure of self-regulation, “the individual's conscious self-control is relatively diminished” (LaRose et al., 2003, p. 232). In this state, individuals are likely to be unable to adapt their behavior to set goals, or this failure may manifest itself in a failure to monitor, judge, or adjust their (digital) behaviors (cf. Soror et al., 2015). van Deursen et al. (2015) found that lower levels of self-regulation are associated with a higher risk of smartphone addiction. This regulatory deficit is of fundamental importance, as it is a mechanism that significantly influences whether excessive use of digital media will develop into problematic or pathological behavior (Tokunaga and Rains, 2010). Conversely, a developed capacity for self-regulation is an essential prerequisite for engaging in self-control. In other words, the more an individual masters self-regulation, the less they need to consciously and deliberately engage in self-control. At the same time, the way in which an individual thinks about the possibility of self-control in general, as well as the individual's beliefs about his or her own control and ability to gain control over his or her own behavior (sense of control) seem to be significant in relation to digital behavior (cf. Shapiro, 2019).

## Methods

### Research objectives and hypotheses

The research goal was to analyze the digital behavior of students in terms of risk (level of screen time, excessive internet use and impulsive DMU) along with their self-regulatory mechanisms (self-control, sense of control and self-regulation of digital media use). Another goal was to verify whether self-control and sense of control predicts the students' degree of self-regulation of digital media use. Two hypotheses were to be verified: (1) Screen time as well as excessiveness and impulsiveness of internet use negatively relate to self-regulation; (2) Self-control and sense of control can predict students' degree of self-regulation of DMU.

### Participants

The research sample included 2,697 students (girls = 1,343; 49.8% and boys = 1,354; 50.2%; average age = 12.9) randomly selected from the second stage of primary schools in the Czech Republic (ISCED level 1, grades 6–9). Participants were drawn from schools located across various urban and rural regions, ensuring geographic diversity. Pupils were selected from intact classes, meaning that classmates may share some environmental influences. The schools represented a range of socioeconomic backgrounds, reflecting typical variability in access to digital technologies and income levels within the country. Data collection was conducted by trained staff using a paper-and-pencil questionnaire. Written informed consent was obtained from all participants and their legal guardians prior to data collection. Participation was voluntary, and all participants were informed about the study's purpose and procedures. All data were collected and analyzed anonymously in accordance with ethical research standards. The study was reviewed and approved by the Ethics Committee of Tomas Bata University in Zlín on March 23, 2023. Data collection took place from October 1 to November 30, 2024.

### Measures

The *Excessive Internet Use Scale* was used to determine how students rate their internet use in the last 6 months. Our assessment is therefore not of an immediate short-term situation, but of a longer period. This tool created by Blinka et al. (2015) consists of five items based on the Griffiths (2005) model of behavioral addictions. The scale includes these items: (1) I have gone without eating and sleeping because of the internet. (2) I have felt bothered when I cannot be on the internet. (3) I have caught myself surfing when I am not really interested. (4) I have spent less time than I should with either family, friends, or doing schoolwork because of the time I spent on the internet. (5) I have tried unsuccessfully to spend less time on the internet. In the original model, excessiveness is measured on a four-point scale ranging from never to very often. In our research, however, we used a five-point scale due to maintain greater compatibility with the five-point scales used in other tools in this research. The Cronbach's coefficient alpha was 0.67.

Items from the *Compulsive Internet Use Scale* (CIUS; Meerkerk et al., 2009) and the *Online Cognition Scale* (OCS; Davis et al., 2002) were adopted to measure *impulsive digital media use*. We used only the items related to impulsive DMU. Three items originally come from CIUS: (1) I often continue to use social media even though I intend to stop. (2) I find it difficult to stop using the internet when I am online. (3) I have been told many times that I spend too much time on my smart phone. One item was adopted from OCS: (1) I stay on my smart phone longer than I intended. In addition, our research team created one item: (1) I am impatient and rush through school assignments to get online. These five items were measured on the five-point scale ranging from “not at all” to “always.” Exploratory factor analysis (EFA) and confirmatory factor analysis (CFA) were conducted to assess the suitability of a single-factor model measuring impulsive digital media use. Factor extraction utilized Maximum Likelihood estimation (cf. Tabachnick and Fidell, 2013). Initial suitability was confirmed by a KMO measure of 0.822 and a significant Bartlett's test (*p* < 0.001). The resulting factor loadings ranged from 0.61 to 0.71, and the single-factor model accounted for 54% of the variance in the items. CFA was used to test the fit of the model. The CFA results are satisfactory (*p* < 0.001; TLI = 0.959; CFI = 0.979; RMR = 0.038; RMSEA = 0.072; PCLOSE = 0.005). Although the *p*-value and *pclose* is less than 0.05, which is probably caused by a large number of the sample, the good values of the fit indices, the strong item-to-item correlations, and the higher factor loadings collectively support our chosen single-factor solution. The Cronbach's coefficient was α = 0.79.

The degree of students' self-control was measured through *The Brief Self-Control Scale* developed by Tangney et al. (2004). Measured on a five-point scale ranging from “not at all” to “very much,” this 13 item scale was designed to determine the behavioral aspects of self-control, which include achievement, impulse control, psychological adjustment, interpersonal relationships, moral emotions and personality. The Cronbach's coefficient alpha was 0.74.

The students' sense of control was measured through four items derived from *The Sense of Control Scale* (Lachman and Weaver, 1998). Measured on a seven-point scale, tool was originally designed for adults and measures a person's sense of control over her or his life in two domains: personal mastery and perceived constraints. We adopted four items measured on the five-point scale ranging from “strongly agree” to “strongly disagree” that were suitable and understandable for students aged 12–15. The items were: (1) There is little I can do to change many of the important things in my life. (2) I often feel helpless in dealing with the problems of life. (3) Other people determine most of what I can and cannot do. (4) What happens in my life is often beyond my control. EFA and CFA were used to assess the suitability of a single-factor model measuring sense of control. Factor extraction was performed using Maximum Likelihood estimation, which was deemed appropriate for the data (cf. Tabachnick and Fidell, 2013). The single-factor solution, which accounted for 47% of the variance in the items, was supported by a KMO value of 0.701 and a significant Bartlett's test (*p* < 0.001). Factor loadings ranged from 0.40 to 0.65. The CFA results indicate a satisfactory fit (*p* < 0.018; TLI = 0.985; CFI = 0.995; RMR = 0.015; RMSEA = 0.033; PCLOSE = 0.842). While the *p*-value is statistically significant, likely due to the large sample size, the very good fit indices, strong item-to-item correlations, and high factor loadings collectively support the chosen single-factor solution. The Cronbach's coefficient alpha was 0.63.

The *self-regulation of digital media use* was measured through a five-item scale designed by Pastor et al. (2022) for evaluating smartphone and internet use regulation strategies. Whereas, the original authors use a four-item scale, we used a five-item scale ranging from “never” to “always” for greater compatibility to five-point scales used in other tools in this research. The scale includes these items: (1) At home, my parents take my phone away when I have other things to do. (2) At home, my parents have to remind me to turn off my phone when I have other things to do. (3) When I have other things to do, I myself turn off the phone, move it away or I disconnect from social media without anyone having to tell me. (4) I check my phone or the internet when I have other things to do. (5) When I am doing things and my phone is off, I cannot stop thinking about what I may be missing out on. The Cronbach's coefficient alpha was 0.61.

To maintain methodological consistency and minimize respondent cognitive load, all measures were converted to a five-point Likert scale. This format was adopted as a balanced compromise between the discriminability of the scales and the clarity of the responses. For this reason, we conducted psychometric analyses to ensure that the modified instruments measure the intended constructs equivalently. Overall, the reliability coefficients were satisfactory, although the attenuated magnitude of some coefficients is noted as a limitation of this study. Furthermore, factor analyses supported the hypothesized dimensionality. The pattern of correlations with other constructs was consistent with theoretical expectations, supporting construct validity of the modified measures.

The instruments were initially translated into Czech by a native speaker. This version was subsequently back-translated into English by a native English speaker. The back-translation was then scrutinized against the original instruments. Based on this comparison, minor adjustments were implemented in the Czech scales. Finally, the comprehensibility and usability of all items were verified using a student sample.

*Screen time* was assessed using two complementary measures. First, participants reported how much time they usually spent using different types of digital devices (smartphone, computer, tablet, gaming console, television) and engaging in various online activities (e.g., social networking, online games, watching videos or films, listening to music, and education) on weekdays and weekends. These self-reported data were used for descriptive purposes to illustrate the structure and distribution of adolescents' digital media use. Second, participants reported their *average daily total screen time* (in minutes per day) based on the value displayed in their smartphone settings (*iOS—Screen Time* or *Android—Digital Balance*). This approach yields a semi-objective, self-reported value derived from device records, which allows for greater accuracy while maintaining feasibility across the sample. These semi-objective total screen time values were used in the main analyses examining relationships between digital behavior and self-regulatory mechanisms. To assess the robustness of associations involving screen time, we conducted a sensitivity analysis using a log-transformed version of screen time. Results remained robust, indicating that extreme values did not drive the observed associations.

### Data analysis

Data were analyzed using IBM SPSS Statistic 28.0. Descriptive statistics were used to determine the level of screen time, relative excessivity of internet use, relative impulsive digital media use, self-control, sense of control and self-regulation of digital media use. Pearson's correlation coefficient was used to determine relationships among the variables. Hierarchical regression analyses were conducted to examine the predictive role of self-control and sense of control on self-regulation of digital media use. Assumptions of linearity, normality, homoscedasticity, and multicollinearity were checked for all models. Residual analysis showed that residuals were approximately normally distributed (M = 0.00, SD = 0.67) and homoscedastic. Standardized residuals ranged from −3.84 to 3.06, with no serious outliers detected, indicating that model assumptions were met. Multicollinearity checks (Tolerance = 0.82, VIF = 1.22) indicated that predictors were not highly correlated. Condition indices (< 12) confirmed the absence of multicollinearity. In the first step (Model 1), self-control and sense of control were entered as predictors. In the second step (Model 2), gender and SES were added as control variables to test the robustness of the main effects. Results indicated that the inclusion of control variables did not meaningfully change the main effects. No missing data were present in the dataset, and all analyses were conducted on complete cases.

## Results

### Screen time, excessive internet use, impulsive digital media use

The average screen time for all students is 4 h and 48 min per day. The standard deviation is high (SD = 3 h and 14 min), however, which indicates a large variance above and below the average value. The median, which we consider the more accurate value in this case, is 4 h and 10 min per day of screen time. A smartphone is the most often used device (average screen time from 3 to 4 h per day). Regarding other devices such as PCs, tablets, gaming consoles and TVs, adolescents indicated less frequent usage (from 0.5 to 1.5 h on average). The most often online activity of adolescents is the use of social networking services (SNS; from 2 to 3 h on average per day). Other frequent activities include watching short videos (e.g., on the YouTube) and films/series as well as listening to music (up to 1.5 h on average).

Three groups of students were identified at the level of the excessive internet use (EIU). The EIU instrument lacks established clinical cutoffs; therefore, we operationalized three groups using standardized scores (z-scores) to determine relative levels of excessive Internet use within the participants. The z-score thus serves as an empirically derived cut-off. In the context of a normal data distribution, this cut-off separates three qualitatively different levels of intensity of use (normal, elevated, and extreme). This approach theoretically aligns with the Problematic Internet Use (PIU) model, which distinguishes between adaptive, maladaptive, and pathological use (cf. Durkee et al., 2016). We deliberately classified participants as non-risk, risk, and high-risk based on z-score thresholds specifically selected to (a) capture the central (majority) portion of the distribution as non-risk, (b) isolate a broader elevated group with a distinctly higher mean EIU score, and (c) specifically identify an extremely high group with a significantly elevated score.

The first group, which may be called *non-risk*, includes 1,527 students (56.6%; boys = 53.2%; girls = 46.8%). The average EIU value of these students is M = 1.54; SD = 0.33 with the average minimum value from 1 to average maximum value to 2 (*z score* ≤ −0.1) on a five-point scale in which the higher the value, the bigger the EIU. The second group, which we termed *risk*, consists of 1,125 students (41.7%; girls = 53.3%; boys = 46.7%). The average EIU for these students is M = 2.71; SD = 0.43, with the average minimum value from 2.20 to average maximum value to 3.80 (*z score* within the interval of 0.2–2.3). The third group, which can be considered as *high-risk*, includes 45 students (1.7%; girls = 62.2%; boys = 37.8%); the average EIU is M = 4.32; SD = 0.32, with the average minimum value from 4 to average maximum value to 5 (*z score* within the interval of 2.60–3.90). While in the *non-risk* group boys outnumber girls, the *risk* and *high-risk* groups are more likely to consist of girls. The excessive internet use is related to screen time at a moderate level (*r* = 0.362; *p* < 0.001), with differences among the three groups of students allowing us to declare this relationship. The median screen time per day of each group is: *non-risk* group = 3 h and 30 min, *risk group* = 5 h and 30 min, *high-risk* group = 8 h and 30 min.

The mean value of the impulsive digital media use (IDMU) is M = 2.47; SD = 0.91 for all students (measured on a five-point scale: the higher the value, the higher the impulsivity). Differences in IDMU can be found among the three groups of students that were identified according to EIU. The *non-risk* group was measured at an average value of IDMU M_IDMU_ = 2.04; SD_IDMU_ = 0.71. The *risk group* showed M_IDMU_ = 3.00; SD_IDTU_ = 0.79 and the *high-risk* group M_IDMU_ = 4.17; SD_IDTU_ = 0.70. The students with higher levels of excessive internet use also indicated higher levels of impulsive digital media use. This finding is supported by correlation analysis, which shows relatively strong correlation between EIU and IDMU (*r* = 0.672; *p* < 0.001). As is the case with EIU, the impulsive digital media use is also related to screen time (*r* = 0.349; *p* < 0.001), indicating that with increased impulsivity, increased screen time can also be expected.

### Self-control, sense of control and self-regulation of digital media use

The level of self-control is M = 3.13; SD = 0.66 for all students (measured on a five-point scale, the higher value, the higher self-control). In the *high-risk* group (students with the highest level of excessive internet use) the average value of self-control is the lowest at M_self − control_ = 2.27; SD_self − control_ = 0.65. In the other two groups, students' self-control is higher: *risk* group M_self − control_ = 2.83; SD_self − control_ = 0.59 and the *non-risk* group much higher at M_self − control_ = 3.37; SD_self − control_ = 0.60.

Similar results were shown regarding the sense of control and self-regulation of digital media use (both factors measured on a five-point scale, the higher value, the higher sense of control and self-regulation of digital media use). Students in the *high-risk* group showed the lowest level of the sense of control and self-regulation of digital media use at M_sense_ _ofcontrol_ = 2.64; SD_senseofcontrol_ = 0.87 and M_self − reg.ofDMU._ = 2.54; SD_self − reg.ofdig.beh._ = 0.65. In the *risk* group, the sense of control and self-regulation of digital media use are higher at M_sense_ _ofcontrol_ = 3.16; SD_senseofcontrol_ = 0.69 and M_self − reg.ofDMU._ = 3.42; SD_self − reg.ofDMU._ = 0.73. Students in the *non-risk* group have the highest level of sense of control at M_senseofcontrol_ = 3.67; SD_senseofcontrol_ = 0.73 and self-regulation of digital media use M_self − reg.ofDMU_ = 3.99; SD_self − reg.ofDMU_ = 0.66.

### Correlation among self-regulatory mechanisms, screen time, excessive internet use and impulsive digital media use

The correlation matrix shows relationships among self-control, sense of control and self-regulation of digital media use (collectively termed here as “self-regulatory mechanisms”) and students' screen time, excessive internet use and impulsive digital media use (Table 1). Generally, self-regulatory mechanisms negatively correlate to screen time, excessive internet use and impulsive digital media use, with the correlation in the interval of *r* = −0.187 to *r* = −0.581 (correlation significant at the 0.001 level). The hypotheses that screen time, excessiveness as well as impulsiveness in internet use negatively relate to self-regulation were all verified. This result indicates that with decreasing self-regulation, students' screen time increases, as do excessive internet use and impulsive digital media use. Further, positive correlations can be seen both among self-regulatory mechanisms (i.e., self-control, sense of control, and self-regulation of digital media use) and among digital media use (i.e., screen time, excessive internet use and impulsive digital media use).

**Table 1:** Correlation among self-regulatory mechanisms, screen time, excessive internet use, and impulsive digital media use (*N* = 2697).

**Variable**	**M**	**SD**	**1**	**2**	**3**	**4**	**5**	**6**
1. Self-control	3.13	0.66	–					
2. Sense of control	3.44	0.77	0.43^**^	–				
3. Self-regulation of digital media use	3.73	0.76	0.46^**^	0.32^**^	–			
4. Screen time	4 h, 48 min.	3 h, 14 min.	−0.28^**^	−0.19^**^	−0.25^**^	–		
5. EUI^a^	2.07	0.74	−0.51^**^	−0.42^**^	−0.49^**^	0.36^**^	–	
6. IDMU^b^	2.48	0.91	−0.53^**^	−0.38^**^	−0.58^**^	0.35^**^	0.67^**^	–

^a^Excessive internet use, ^b^impulsive digital media use, ^**^p < 0.001.

### Self-control and sense of control predict self-regulation of digital media use

The hypothesis that self-control and sense of control can predict students' degree of self-regulation of digital media use was verified through regression analysis (Table 2). Self-control proved to be a stronger predictor of self-regulation of digital media use (β = 0.40; *p* < 0.001) than sense of control (β = 0.15; *p* < 0.001).

**Table 2:** Self-control and sense of control as predictors of self-regulation of digital media use.

**Variables**	**B**	**SE**	***t***	**95% CI**		***p***	***R^2^***	**SEE**	***F***	***p***
				**LL**	**UL**					
(Constant)	1.797	0.071	25.232	1.657	1.936	< 0.001				
Self-control	0.457	0.021	21.272	0.415	0.499	< 0.001				
Sense of control	0.145	0.018	7.861	0.109	0.182	< 0.001				
							0.229	0.666	402.000	< 0.001

Including gender and perceived SES as covariates did not meaningfully change the results (see text for details).

To verify the robustness of these findings, gender and perceived SES were added as control variables in a hierarchical regression. The inclusion of these covariates slightly increased the explained variance (*R*^2^ increased from 0.230 to 0.235), while the coefficients for self-control and sense of control remained virtually unchanged and statistically significant. Gender (β = 0.057, *p* < 0.001) and perceived SES (β = 0.036, *p* = 0.035) had small but significant effects. Self-control and sense of control explain 23% of variability of self-regulation of digital media use, thus these predictors have a relatively high potential to influence the self-regulation of digital media use.

## Discussion

The current study aimed to extend the conceptualization of adolescents' digital media use by examining the relationships between self-regulatory mechanisms—specifically trait self-control, sense of control, and self-regulation of digital media use—and risky patterns of digital media use. Risky use was conceptualized as excessive and impulsive engagement in digital activities that could interfere with daily functioning. The study also considered screen time as a potential risk factor and analyzed the extent to which trait self-control and sense of control predict domain-specific self-regulation. Our findings highlighted the variability in adolescents' digital media use associated with self-regulatory mechanisms and suggested that general self-control can partially account for domain-specific self-regulation in the digital context.

Several noteworthy findings emerged. First, excessive internet use and impulsive digital media use were strongly interrelated, suggesting that these forms of risky behavior often co-occur. Second, both self-control and sense of control significantly predicted domain-specific self-regulation of digital media use, indicating the combined role of trait-level and context-specific aspects of self-regulation. Third, adolescents in higher-risk groups showed lower levels of both self-control and sense of control, as well as lower domain-specific self-regulation, suggesting that reduced self-regulatory capacities are consistently associated with more risky digital media use. Fourth, screen time was moderately associated with risky digital media use but showed considerable variability among adolescents, indicating that the duration of media use represents only one aspect of digital media engagement.

Taken together, these findings should be interpreted in the context of adolescence as a developmental period characterized by an imbalance between motivational systems and cognitive control (Steinberg, 2010). This imbalance makes adolescents more susceptible to immediate gratification and peer influence, factors that are particularly pronounced in digital environments (da Silva Pinho et al., 2024; Montag et al., 2024). Consequently, the boundary between adaptive and risky digital media use may become less distinct, especially among individuals with weaker self-regulatory mechanisms (Tangney et al., 2004; Li et al., 2021). These results highlight the importance of considering both general self-regulatory traits and context-specific mechanisms when analyzing risky digital media behaviors.

Adolescents in the present sample spent a considerable portion of their day on digital media, predominantly on smartphones, with social networking and short videos being the most frequent activities. This pattern aligned with contemporary research indicating that mobile devices are central to adolescents' daily digital behavior and that social media platforms provide highly engaging, socially reinforcing content (Przybylski and Weinstein, 2019; Montag et al., 2024).

Stratifying adolescents into groups based on the risk of excessive internet use revealed marked heterogeneity in digital engagement. Median daily screen time progressively increased from approximately 3.5 h in the non-risk group to over 8 h in the high-risk group, indicating that more intensive excessive internet use is associated with longer online engagement. In the context of the Czech Republic, overall adolescent digital engagement appears lower than in many other countries (Boniel-Nissim et al., 2022); although recent observations suggest an increasing trend in problematic digital media use, with most adolescents maintaining balanced use, but a minority showing elevated and potentially problematic patterns (Zdravagenerace.cz, 2023).

Time spent using new media, particularly on smartphones, has long been a topic of research, with mixed evidence regarding its impact on adolescents' psychosocial functioning (Cho et al., 2023). In studies examining problematic internet use (PIU) or smartphone addiction, screen time often emerges as a factor associated with higher risk (Niskier et al., 2024; Muppalla et al., 2023; Randjelovic et al., 2021). However, empirical findings indicate that this association is generally weak, and screen time alone does not provide a clear or consistent indicator of problematic use (Tomczyk and Lizde, 2023). In our study, screen time showed moderate associations with excessive and impulsive digital media use. This indicates that the sheer duration of engagement with digital media is only one aspect of digital behavior and needs to be considered alongside the quality, context, and type of activities (Caplan and High, 2006; Hou et al., 2019; Twenge and Farley, 2021).

Impulsivity represents an important dimension of individual differences in adolescents' digital behavior. Our data indicated that excessive internet use and impulsive digital media use were closely related dimensions of adolescents' behavior, with students in the high-risk group for excessive internet use exhibiting higher levels of impulsive digital media use. This pattern highlights how impulsive tendencies often co-occur with excessive digital media use, with the intensity of these behaviors potentially varying between different user groups (Cao et al., 2007; Salvarli and Griffiths, 2022; Wegmann et al., 2020; Beard and Wolf, 2001; Billieux et al., 2015; Simşek et al., 2019).

Some studies indicate that impulsive behavior is not automatically associated with problematic digital media use for all individuals. Impulsive behavior becomes problematic when it occurs in an inappropriate context and the individual cannot exert control over it (Turel and Qahri-Saremi, 2016). Reduced self-control is consistently associated with higher levels of excessive and impulsive digital media use, representing a potential risk for problematic digital behavior (Doebel et al., 2017; Blinka et al., 2015; cf. Kuss and Pontes, 2019; Wang et al., 2023; West et al., 2021). The results of our study confirmed that adolescents with lower self-control exhibited both excessive internet use and impulsive digital media use. In higher-risk groups for excessive internet use, self-control was lowest and impulsivity highest, indicating a significant link between weakened self-regulatory mechanisms and more risky digital media use. Individuals with lower self-control are likely to respond more impulsively to enticing stimuli, show a stronger tendency to succumb to immediate rewards, and find it harder to resist social or emotional triggers. Conversely, higher self-control may act as a psychological protective factor, enabling adolescents to delay gratification and interrupt automatic patterns of digital behavior (Tangney et al., 2004).

At the same time, it cannot be assumed that general (trait) self-control is expressed uniformly across all behavioral domains. In other words, high general self-control is not automatically associated with stronger domain-specific self-regulation in digital contexts. Research focusing on domain specificity in self-control suggests that self-control is not a purely general trait but tends to vary across behavioral domains (Duckworth and Tsukayama, 2015). Our regression analysis confirmed that general self-control partially explained the variability in domain-specific digital media self-regulation, but it alone did not provide a complete explanation. In addition to general self-control, the sense of control also contributed to explaining variability in domain-specific self-regulation, with adolescents reporting a higher sense of control being more capable of regulating their behavior while using digital media. However, even together, these two predictors accounted for only part of the observed variance, suggesting that domain-specific self-regulation may also be influenced by other situational and motivational factors. This finding aligns with the theory of domain-specific self-control (Duckworth and Tsukayama, 2015), which argues that self-control is not a uniform, universal trait but varies depending on the specific domain of behavior, in this case, digital behavior. While general self-control may contribute to domain-specific self-regulation, it is not the sole factor. As our results show, the sense of control, which is associated with momentary motivational factors, appears to be related to regulating digital behavior. This also corresponds with the principles of the hot/cool system theory (Metcalfe and Mischel, 1999), which suggests that domain-specific self-regulation emerges from the interaction between stable personality traits (cool system) and momentary sources of motivation and control (hot system).

In the literature, the sense of control is associated with better adaptation, lower vulnerability to stress, and a reduced likelihood of developing addictive behaviors (Brailovskaia and Margraf, 2022; Horwood and Anglim, 2021). In the digital context, this construct may reflect the extent to which adolescents perceive they can control their behavior despite pressures from the online world, such as algorithmic prompts, social comparison, or notifications. Our results revealed that a higher sense of control was associated with lower impulsivity in digital media use and less excessive internet use, with adolescents in the high-risk group for excessive internet use reporting a lower sense of control than those in the low-risk group. Correlation analyses further revealed moderate to strong associations between sense of control, self-control, and domain-specific self-regulation, confirming the importance of the sense of control for regulating digital behavior.

Overall, our findings indicate that adolescents' self-regulatory mechanisms involve general self-control (trait self-control), domain-specific self-regulation, and the sense of control, all of which are related to risky digital media use. Low self-control, a low sense of control, or weaker domain-specific self-regulation were associated with higher impulsivity and excessive media use. These findings support the idea that effective self-regulation is not merely a matter of willpower but a broader system encompassing motivational, cognitive, and emotional processes. Enhancing the sense of control and self-regulation could therefore help adolescents better manage digital temptations. However, future longitudinal and experimental research is necessary to verify whether improvements in self-control or sense of control actually lead to better regulation of digital media use.

This study has several limitations that should be considered when interpreting the findings. First, we relied on self-report measures, which may be subject to recall bias, social desirability effects, or subjective interpretation. While this approach can introduce some bias, it remains the most feasible method for assessing multiple constructs in a large adolescent sample and allowed us to capture subjective experiences such as perceived self-control and sense of control, which cannot be directly measured and must be reported by participants themselves. Second, some scales demonstrated moderate internal consistency. Although this may have slightly attenuated the observed associations due to measurement error, the coefficients remained within the acceptable range for studies on complex psychological constructs in younger populations (Nunnally and Bernstein, 1994), and the main relationships remained statistically significant, suggesting that the findings are robust. Third, the cross-sectional design precludes any causal inference; while regression analyses revealed significant associations between self-control, sense of control, and digital media self-regulation, the direction of effects cannot be established. Finally, although the sample was large and nationally representative, cultural and contextual factors may influence digital behavior, limiting the generalizability of findings to adolescents in other countries (Odgers and Jensen, 2020). Specific aspects of digital behavior, such as average screen time or the ways adolescents engage with digital devices, are likely influenced by country-specific factors, including educational practices, cultural norms, and availability of technology. Nonetheless, the observed associations among self-control, sense of control, and digital media self-regulation likely reflect underlying psychological mechanisms—such as executive functioning and self-regulatory capacity—that are relevant across cultures (Trommsdorff, 2009). Future research should investigate these relationships in other countries to assess cross-cultural generalizability.

These findings have both theoretical and practical implications. From a theoretical perspective, they support integrative models of digital behavior, which suggest that risky digital media use is best understood as the result of the combined influence of individual differences and contextual factors (e.g., digital environment; Baumeister and Vohs, 2007; Reinecke et al., 2021). Furthermore, they show that self-regulation is domain-specific, meaning that general self-control alone is insufficient to fully explain adolescents‘ adaptive digital behavior. Instead, the interaction between general self-control and perceived sense of control appears to be a key factor in shaping digital behavior. This perceived sense of control, which is subjective, is important for how individuals evaluate their ability to regulate their own behavior in the digital environment. The findings suggest that both individual self-regulatory capacity and subjective assessment of control are linked to differences in adolescents' behavior in digital contexts, which may open new avenues for designing digital behavior interventions.

From a practical perspective, the results highlight the importance of interventions and prevention programs that strengthen both general and domain-specific self-regulation, rather than merely limiting screen time. Programs that promote adolescents' sense of autonomy and encourage reflective awareness of media habits (e.g., mindfulness, digital literacy training) may be particularly effective in fostering adaptive digital behavior. Additionally, the findings underscore the value of tiered interventions that are tailored to the level of risk faced by adolescents. Adolescents exhibiting high levels of risky digital media use may benefit from more intensive, individualized strategies aimed at enhancing both general self-control and domain-specific regulatory skills. In contrast, adolescents at lower risk may benefit from universal preventive measures, such as self-regulation exercises integrated into educational activities, guided reflection on media behavior, or peer-led initiatives that encourage healthy digital habits. Tailoring interventions to match adolescents‘ risk levels can optimize support for adaptive digital behavior and help prevent impulsive or excessive use of digital media.

## Conclusion

Overall, the study showed that domain-specific self-regulation of digital media use was closely associated with general trait self-control and the subjective sense of control. Adolescents with lower levels of these self-regulatory mechanisms exhibited higher levels of risky digital media behavior, including excessive and impulsive use. These mechanisms accounted for a moderate portion of the variance in digital self-regulation, suggesting that individual differences in control processes play an important role in understanding adolescents' risky digital media behavior. The findings highlight digital media self-regulation as a multi-layered construct, linked to both general trait self-control and domain-specific self-regulatory mechanisms and provide a basis for targeted support strategies to identify adolescents who may benefit from interventions aimed at enhancing self-control and the sense of control, ultimately promoting healthier and safer engagement with digital media.

## Data Availability

The raw data supporting the conclusions of this article will be made available by the authors, without undue reservation.
